# Fibroblast growth factor‐2/platelet‐derived growth factor enhances atherosclerotic plaque stability

**DOI:** 10.1111/jcmm.14850

**Published:** 2019-11-21

**Authors:** Yang Mao, Xiao Qiong Liu, Yu Song, Chun Gang Zhai, Xing Li Xu, Lei Zhang, Yun Zhang

**Affiliations:** ^1^ Department of Cardiology The Key Laboratory of Cardiovascular Remodeling and Function Research Chinese Ministry of Education Chinese National Health Commission and Chinese Academy of Medical Sciences The State and Shandong Province Joint Key Laboratory of Translational Cardiovascular Medicine Qilu Hospital of Shandong University Jinan China; ^2^ Department of Cardiology Shanghai Institute of Cardiovascular Diseases Zhongshan Hospital Fudan University Shanghai China

**Keywords:** atherosclerosis, fibroblast growth factor (FGF‐2), pericytes, platelet‐derived growth factor‐BB (PDGF‐BB), vascular normalization

## Abstract

Increased immature neovessels contribute to plaque growth and instability. Here, we investigated a method to establish functional and stable neovessel networks to increase plaque stability. Rabbits underwent aortic balloon injury and were divided into six groups: sham, vector and lentiviral transfection with vascular endothelial growth factor‐A (VEGF)‐A, fibroblast growth factor (FGF)‐2, platelet‐derived growth factor (PDGF)‐BB and FGF‐2 + PDGF‐BB. Lentivirus was percutaneously injected into the media‐adventitia of the abdominal aorta by intravascular ultrasound guidance, and plaque‐rupture rate, plaque‐vulnerability index and plaque neovessel density at the injection site were evaluated. Confocal microscopy, Prussian Blue assay, Evans Blue, immunofluorescence and transmission electron microscopy were used to assess neovessel function and pericyte coverage. To evaluate the effect of FGF‐2/PDGF‐BB on pericyte migration, we used the mesenchymal progenitor cell line 10T1/2 as an in vitro model. VEGF‐A‐ and FGF‐2‐overexpression increased the number of immature neovessels, which caused intraplaque haemorrhage and inflammatory cell infiltration, eventually resulting in the plaque vulnerability; however, FGF‐2/PDGF‐BB induced mature and functional neovessels, through increased neovessel pericyte coverage. Additionally, in vitro analysis of 10T1/2 cells revealed that FGF‐2/PDGF‐BB induced *epsin‐2* expression and enhanced the VEGF receptor‐2 degradation, which negatively regulated pericyte function consistent with the in vivo data. These results showed that the combination of FGF‐2 and PDGF‐BB promoted the function and maturation of plaque neovessels, thereby representing a novel potential treatment strategy for vulnerable plaques.

## 1 INTRODUCTION

Acute cardiovascular events, because of rupture or erosion of an atherosclerotic plaque, represent the major cause of morbidity and mortality in atherosclerotic patients; hence, there is an emerging need for new therapies to stabilize atherosclerotic lesions. Growing evidence suggests that plaque neovessels are important contributors to plaque destabilization and rupture because of their inability to mature properly, resulting in compromised integrity and leaky vascular walls.^1^, ^2^, ^3^, ^4^ Therefore, intraplaque neovascularization might represent a novel therapeutic target in advanced atherosclerosis.

Newly formed microvessels are unable to mature properly because of an imbalance between pro‐angiogenic and pro‐maturation factors,^4^ which cause leaky neovessels that form an entrance for erythrocytes, lipids and inflammatory factors. A central pathway coordinating pro‐angiogenesis is that of vascular endothelial growth factor (VEGF)‐A, which pre‐clinical studies indicate as a potential treatment for tumours and peripheral artery disease; however, large‐scale clinical trials using VEGF‐A in tumours and patients with peripheral artery disease have proven disappointing.^5^ Uncertainty regarding whether the inhibition of plaque neovessel results in stable plaques remains largely because of the lack of an appropriate plaque‐neovessels animal model, which introduces discrepancies in the outcomes of several studies and complicates science‐based conclusions.^6^

The development of atherosclerotic lesions involves three different pathoanatomic stages of angiogenesis: initiation, progression and complication. Studies of the initiation phase using a rabbit model fed an atherogenic diet for 3 weeks showed that local delivery of bevacizumab (a monoclonal antibody against VEGF‐A) inhibited plaque angiogenesis, resulting in smaller atherosclerotic plaques.^6^, ^7^ Despite their clinical relevance, there are few studies of anti‐angiogenic therapy on the progression and complication stages and inappropriate anti‐angiogenic therapy at these two stages can potentially cause hypoxia and induce endothelial apoptosis, resulting in the loss of integrity of the endothelial vessel lining (bleeding).^8^, ^9^ This observation raised concerns regarding the importance of vascular‐normalization therapy in atherosclerotic plaque.^10^ Vasculature normalization is a strategy, whereby angiogenic growth factors improve neovessel perivascular‐cell coverage and maturity.^11^ Therefore, elucidating of the mechanisms and signalling pathways underlying vascular‐normalization pathways is critical.

Fibroblast growth factor (FGF)‐2 is a potent growth factor that stimulates endothelial‐cell migration and proliferation and encourages mitogenesis of smooth muscle cells (SMCs).^12^ The platelet‐derived growth factor‐BB (PDGF‐BB)/PDGF receptor‐β (PDGFR‐β) axis is the best‐characterized signalling system for perivascular‐cell recruitment.^13^ Previous studies indicate that the co‐existence of two angiogenic factors, even at low levels, could have a greater functional impact than high levels of each alone^14^; therefore, using FGF‐2 and PDGF‐BB together might have profound implications for plaque neovessel normalization.

Here, we propose a new strategy for local delivery of FGF‐2 and PDGF‐BB lentivirus into atherosclerotic plaque in order to induce stable and long‐lasting expression of growth factors and ‘normalize’ the abnormal structure and function of neovessels, thereby improving plaque stability.

## 2 MATERIALS AND METHODS

### 2.1 Ethics statement

A total of 90 male New Zealand white rabbits (1.7‐2.1 kg) were randomly selected and housed at the Animal Care Center of Shandong University Qilu Hospital (Jinan, China). Animal experiments complied with the Animal Management Rule of the Ministry of Public Health, People's Republic of China (documentation 55, 2001), and the animal experiments conform to the animal ethics and animal welfare (animal ethics audit: DWLL‐2016‐022). The experimental protocol was approved by the Animal Care Committee of Shandong University.

### 2.2 Experimental protocol

**Figure 1:**
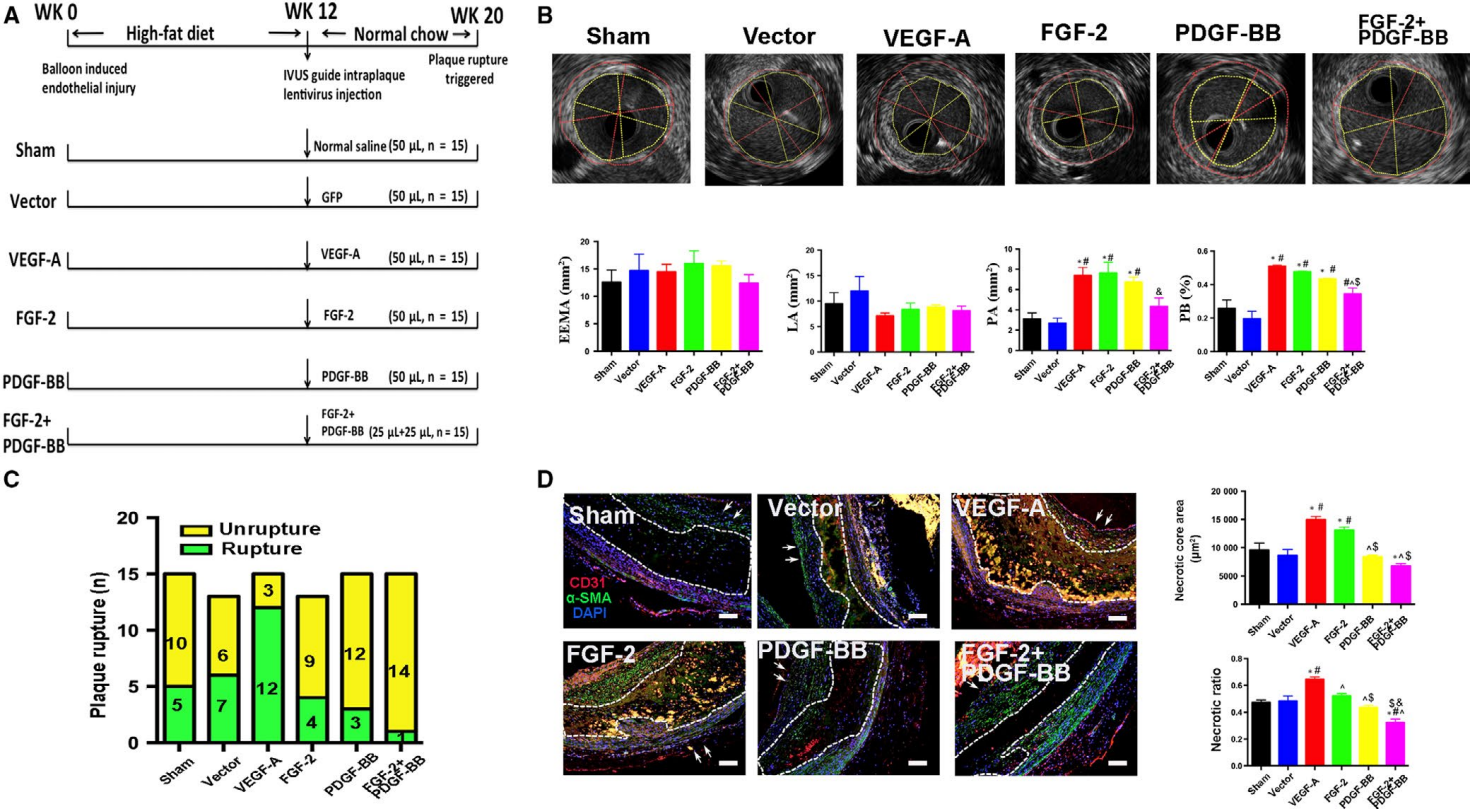
General condition of rabbits. A, Animal grouping and time course for the in vivo studies. B, IVUS images and measurement of external elastic membrane area (EEMA), lumen area (LA), plaque area (PA) and plaque burden (PB) (mean ± SEM; n = 3). Red line indicated the area of EEMA. Yellow line indicated the area of LA. C, Bars indicate the number of animals with IPH and plaque rupture in the sham (5/15), vector (6/13), VEGF‐A (12/15), FGF‐2 (9/13), PDG‐BB (3/15) and FGF‐2 + PDGF‐BB (1/15) groups. D, Representative images of the necrotic core and quantification of necrotic core area and necrotic ratio in rabbit atherosclerotic plaques (mean ± SEM; n = 5). Bar, 20 μm. Arrow indicated lumen surface. ^*^ *P* < .05 vs Sham; ^#^ *P* < .05 vs Vector; ^^^ *P* < .05 vs VEGF‐A; ^$^ *P* < .05 vs FGF‐2; ^&^ *P* < .05 vs PDGF‐BB

A rabbit model of atherosclerotic plaque was generated according to our previous study with minor modifications.^15^ After anesthetization with 3% pentobarbital sodium (30 mg/kg, intravenous), all 90 rabbits underwent balloon‐induced endothelial injury in the abdominal aorta and were subsequently fed a high‐cholesterol diet (1% cholesterol) for 12 weeks. Thereafter, the hypercholesterol diet was replaced with normal chow, and the rabbits were randomly divided into six groups for treatment: sham (control group; no treatment), vector, VEGF‐A, FGF‐2, PDGF‐BB and FGF‐2 + PDGF‐BB (n = 15 rabbits/group) (Figure 1A).

At week 12 and identification of the injected plaque in the abdominal aorta via intravascular ultrasound imaging (IVUS), an incision (5‐7 cm) was made medially in the abdomen, and the plaque was isolated. Using IVUS observation, a 50‐μL suspension of lentivirus encoding green fluorescent protein (GFP), VGF‐A (titre 1 × 10^9^ UT/mL), FGF‐2 (titre 1 × 10^9^ UT/mL), PDGF‐BB (titre 1 × 10^9^ UT/mL) or FGF‐2 + PDGF‐BB (titre 1 × 10^9^ UT/mL; Shanghai GenePharma Co., Ltd) (Table S1) was injected from the aortic adventitia into plaques in the abdominal aorta in all groups, except sham. Injection sites were marked by an iliopsoas stitch, and the distance from each site to the bifurcation of the iliac artery was recorded (Figure S1A,B).^16^ Eight weeks after transfection, plaque disruption was triggered by intraperitoneal administration of Russell's viper venom (Sigma‐Aldrich; 0.15 mg/kg) and intravenous injection of histamine (0.02 mg/kg) 30 minutes later.^16^ Before euthanization, three rabbits from each group were injected intravenously with 1% Evans Blue dye. All rabbits were killed, and a 1‐cm long section of the aorta was harvested from the injected segments. The bodyweight of all rabbits was monitored throughout the experiment.

### 2.3 IVUS assay

Each IVUS experiment was performed according to a standard procedure. IVUS imaging was conducted using a 3.2‐F catheter equipped with a 40‐MHz single‐rotating‐element transducer connected to an IVUS system (Galaxy; Boston Scientific). The catheter was withdrawn to the abdominal aorta by a motorized pullback device and at a constant speed of 0.5 mm/s.^16^

### 2.4 Blood lipid analysis

Blood samples were centrifuged at 1,765.38 *g* for 15 minutes at 4°C, and serum samples were collected and stored at −80°C. Serum levels of total cholesterol, triglycerides, low‐density lipoprotein cholesterol and high‐density lipoprotein cholesterol were measured by enzymatic assays using an automated biochemical analyzer (Roche Hitachi917; Block Scientific).

### 2.5 Histopathology and immunohistochemistry (IHC)

The abdominal aorta (1‐cm long) was fixed in 4% formaldehyde for 24 hours, and 5‐mm‐thick segments were then serially sectioned. Frozen sections were stained with Oil Red O (Sigma‐Aldrich) to determine the lipid content, and paraffin sections were subjected to Sirius Red, haematoxylin and eosin (H&E) and IHC staining, respectively. Immunohistochemistry staining was performed using standard techniques, as described previously.^17^ Briefly, endogenous peroxidase activity was inhibited by incubation with 3% hydrogen peroxide and sections were blocked with 5% bovine serum albumin and incubated for 12 hours at 4°C with primary antibodies. After washing with phosphate‐buffered saline (PBS), sections were incubated with secondary antibodies at 37°C for 20 minutes. Immunohistochemistry staining results were analysed using a diaminobenzidine kit (Zhongshan Goldenbridge Biotechnology), and haematoxylin was used to counterstain the nucleus. The primary antibodies used included mouse anti‐rabbit regulation of Ace2 and morphogenesis (RAM)‐11 (M063301‐8; Dako Glostrup, Denmark); α‐SMC actin (HPA014539; Sigma‐Aldrich) and CD31 (ab9498; Abcam). The cross‐reactivity between rabbit antigens and primary antibodies was tested in preliminary experiments (data not shown) and confirmed by negative‐control experiments involving non‐immune IgG instead of primary antibodies. Histopathologic slides were analysed using Image‐Pro Plus 6.0 (v 6.0; Media Cybernetics). The area of positive IHC staining was expressed as the proportion of the stained area divided by the total plaque area in at least five high‐power fields. Five high‐power fields in five plaques from each rabbit were selected for quantitative measurement and averaging. The vulnerability index was calculated as follows: (macrophage staining % + lipid staining %)/(SMC % + collagen fibre %).^17^ Five random high‐power fields were selected for each sample in order to quantify the microvessel density in CD31‐stained sections, and then, the microvessels were quantified by the plaque area.

### 2.6 Immunofluorescence (IF) staining

Immunohistochemistry staining was performed using standard techniques, as described previously.^17^ Sections were incubated with VEGF‐A (ab1316; Abcam), FGF‐2 (ab181; Abcam), FGF receptor (FGFR)‐1 (ab10646; Abcam), FGFR‐2 (ab10648; Abcam), PDGF‐BB (ab178409; Abcam), PDGFR‐β (3169; Cell Signaling Tchonology), CD31 (ab9498, ab222783; Abcam), neuron‐glial antigen 2 (NG2, ab129051; Abcam,), anti‐glycophorin A (ab194397; Abcam,) and α‐SMC actin (ab7817, Abcam; 19245, Cell Signaling Tchonology) antibody at 4°C. Alexa Fluor 594 secondary antibody (anti‐rabbit IgG, 8889S), Alexa Fluor 555 secondary antibody (antimouse IgG, 4409S), Alexa Fluor 488 secondary antibody (antimouse IgG, 4408S; anti‐rabbit IgG, 4412S) and DAPI were used. Images were visualized by laser scanning confocal microscopy (LSM710; Zeiss).

### 2.7 Evans Blue permeability assay

Evans Blue dye (2%; 2 mL/kg) was injected into the ear‐vein of rabbits, and 1 hour after the injection, rabbits were killed and perfused with PBS through the left ventricle to clear the free dye from the vascular volume. Abdominal aorta plaques were removed, dried at 60°C overnight and weighed before Evans Blue extraction using formamide (1 mL/100 mg) at 37°C for 16 hours. Evans Blue was quantified by spectrometry at 620 nm (EMAX Plus Microplate Reader; Molecular Devices).

### 2.8 Prussian Blue staining

Iron‐positive hemosiderin deposits within complicated plaques were quantified using Prussian Blue staining.^18^ Paraffin sections were dewaxed, rehydrated and washed with PBS three times, followed by the addition of 5 wt% potassium ferrocyanide and 10 vol% HCl and incubation at room temperature for 20 minutes before washing with distilled water three times. Images were acquired under an Olympus BX51 microscope (Olympus).

### 2.9 Transmission electron microscopy (TEM)

Abdominal aorta plaque segments from the Sham, GFP, VEGF‐A, FGF‐2, PDGF‐BB and FGF‐2 + PDGF‐BB groups were fixed in 2% glutaraldehyde/4% paraformaldehyde in sodium cacodylate buffer overnight at 4°C and processed for TEM as described previously.^19^

### 2.10 Cell culture

b.END3 and 10T1/2 cells were kindly provided by Stem Cell Bank, Chinese Academy of Science (Beijing, China). Cell monolayers were incubated in Dulbecco's modified Eagle's medium containing 10% foetal bovine serum and 2 mmol/L glutamine in 5% CO_2_ and 95% humidified air at 37°C.

### 2.11 Cell‐migration assay

Pericytes migration ability was monitored using a wound‐healing assay. Treated pericytes were wounded using a yellow tip, detached cells were removed, and fresh low‐serum medium containing FGF‐2 (50 ng/mL) or PDGF‐BB (10 ng/mL) was added. In some experimental settings, pericytes were incubated for 48 hours with 50 ng/mL FGF‐2 in a 6‐well plate prior to stimulation with PDGF‐BB (10 ng/mL). Images were captured at 0 and 6 hours after scratching, and wound closure was analysed using of Image‐Pro Plus (v.6.0; Media Cybernetics).

### 2.12 Transwell migration assay

Chemotactic migration of pericytes was analysed using modified Boyden chambers. Pericytes (5 × 10^4^) were seeded in the upper chamber and FGF‐2 (50 ng/mL) or PDGF‐BB (10 ng/mL) in Dulbecco's modified Eagle medium containing 2% foetal bovine serum was placed in the lower chambers. In some experimental settings, pericytes were incubated for 48 hours with 50 ng/mL FGF‐2 in a 6‐wells plate prior to stimulation with PDGF‐BB (10 ng/mL). Cells were allowed to migrate for 12 hours (n = 5). Non‐migrated cells were removed, and migrated cells on the lower side of the membrane were stained with Crystal Violet. Migrated cells were captured in five random fields.

### 2.13 Transient transfection

Pericytes were transfected for 8 hours with small interfering (si) RNAs directed against *epsin‐2* (GenePharma, Shanghai, China) using Lipofectamine 2000 (Thermo Fisher Scientific Waltham, MA, USA) according to manufacturer protocol. Scrambled non‐targeting control siRNA was used as a negative control.

### 2.14 Cell‐recruitment assay

Cell‐recruitment assays were performed, as previously described.^20^ bEND.3 (4 × 10^5^) and 10T1/2 (2 × 10^5^) cells, labelled with PKH26 and carboxyfluorescein succinimidyl ester, respectively, were co‐cultured in Matrigel‐coated 24‐well plates, and at 6 hours post‐stimulation with FGF‐2 (50 ng/mL) or PDGF‐B (20 ng/mL), and images were obtained using a microscope (IX71‐SIF; Olympus, Japan). In some experimental settings, pericytes were incubated for 48 hours with 50 ng/mL of FGF‐2 in a 6‐well plate prior to stimulation with PDGF‐BB (10 ng/mL). Quantification was performed by counting the number of attached bEND.3‐10T1/2 cells from five high‐power fields in three independent experiments.

### 2.15 Quantitative reverse transcription‐polymerase chain reaction (qRT‐PCR)

Total RNA was extracted from tissue samples using Trizol reagent (Invitrogen), and the IQ5 real‐time PCR thermocycler (Bio‐Rad) was used to perform the qRT‐PCR. mRNA levels of *VEGF‐A*, *FGF2* and *PDGF‐BB* were quantified with SYBR Green qPCR master mix reagent (Takara Biotechnology). The results were normalized against *glyceraldehyde 3‐phosphate dehydrogenase* expression and calculated using the 2^−ΔΔCT^ method. Primers are listed in Table S2.

### 2.16 Western blot analysis

Proteins were extracted from rabbit aortas and cell lysates, and tissues and cells were lysed in lysis buffer (100 mmol/L Tris‐HCl [pH 6.8], 4% [m/v], sodium dodecyl sulphate, 20% [v/v] glycerol, 200 mmol/L β‐mercaptoethanol, 1 mmol/L phenylmethylsulfonyl fluoride and 1 g/mL aprotinin). Proteins were transferred to polyvinylidene fluoride membranes (0.45 mm; Millipore) and incubated overnight at 4°C with primary antibodies for FGFR‐2 (ab10648; Abcam), VEGFR‐2 (2479; Cell Signaling Technology), FGFR‐1 (M19B2; Novus Biologicals), PDGFR‐β (ab69506; Abcam), p‐VEGFR‐2 (Tyr1175; 2478; Cell Signaling Technology), p‐PDGFR‐β (Tyr1009; 3124; Cell Signaling Technology), hypoxia‐inducible factor (HIF)‐1α (ab16066; Abcam), epsin‐1 (ab232764; Abcam), epsin‐2 (NBP2‐16359; Novus Biologicals), β‐actin (3700; Cell Signaling Technology) and GAPDH (51332; Cell Signaling Technology). Protein bands on the membrane were visualized using chemiluminescence (Millipore) and quantified by densitometry.

### 2.17 Statistical analysis

All numerical data were expressed as the means ± standard error of the mean (SEM), and all analyses were performed using SPSS (v.20.0; IBM Corp.). Differences in continuous variables among multiple groups were evaluated using analysis of variance followed by the least squares difference test (with equal variances assumed) or Dunnett's T3 test (equal variances not assumed). A *P* < .05 was considered statistically significant.

## 3 RESULTS

### 3.1 General condition of the animal model

Ninety rabbits received media‐adventitia injection surgery (Figure 1A), with 86 rabbits showing full recovery without complications. Two rabbits from the FGF‐2 group died 5 days after surgery from postoperative infection, and two rabbits from the vector group died of diarrhoea 12 days after surgery. Rabbits from different groups did not differ in bodyweight or serum lipid profiles (Table S3).

Before the formal tests, we performed a pre‐test to verify the exact time‐point at which the lentiviral effect on the plaques could be observed. Frozen sections corresponding to 8 weeks post‐GFP lentiviral injection showed significant expression of green fluorescence within the plaque (Figure S2A). The viruses were efficiently expressed within the plaques as tested by RT‐PCR (Figure S2B‐C), and the serum levels of the growth factors were not different among different groups (Figure S2E‐G). According to the location of cell distribution and the colour of staining, VEGF‐A, FGF‐2 and PDGF‐BB were all widely expressed in various cell types such as endothelial cells, smooth muscle cells and macrophages. However, different from the control groups (sham and vector), VEGF‐A and FGF‐2 produced by injected viruses in the intervened groups were mainly expressed in macrophages, whereas PDGF‐BB produced by injected viruses was mainly expressed by smooth muscle cells (Figure S2H).

All rabbits were examined by IVUS before euthanasia. The lumen area and external elastic membrane area in the abdominal aorta did not differ among the six groups (*P* > .05) (Figure 1B); however, the plaque area and plaque burden values were higher in groups VEGF‐A and FGF‐2 than in groups sham and vector (*P* < .05) (Figure 1B).

### 3.2 Combined treatment with FGF‐2 and PDGF‐BB protects plaques from rupture

We then investigated the effect of FGF‐2 + PDGF‐BB on atherosclerotic plaque stability. We triggered plaque disruption by intraperitoneal injection of Russell's viper venom and histamine, and pathological analysis confirmed that gross haemorrhage and extensive necrosis occurred in 12 of 15 rabbits treated with VEGF‐A lentivirus. Of 13 rabbits treated with FGF‐2 lentivirus, nine suffered from disruption and thrombosis after triggering; however, only three and one rabbit from groups PDGF‐BB (n = 15) and FGF‐2 + PDGF‐BB (n = 15), respectively, exhibited these conditions. Moreover, the rate of plaque rupture at the site of injection was significantly lower in the FGF‐2 + PDGF‐BB group than in the sham (five rabbits; n = 15) and vector groups (six rabbits; n = 13) (Figure 1C). Additionally, the necrotic core size of plaques was markedly diminished in the FGF‐2 + PDGF‐BB group, further supporting the protective role of FGF‐2 + PDGF‐BB on plaque stability (Figure 1D).

### 3.3 Combined FGF‐2 and PDGF‐BB enhances plaque stability

**Figure 2:**
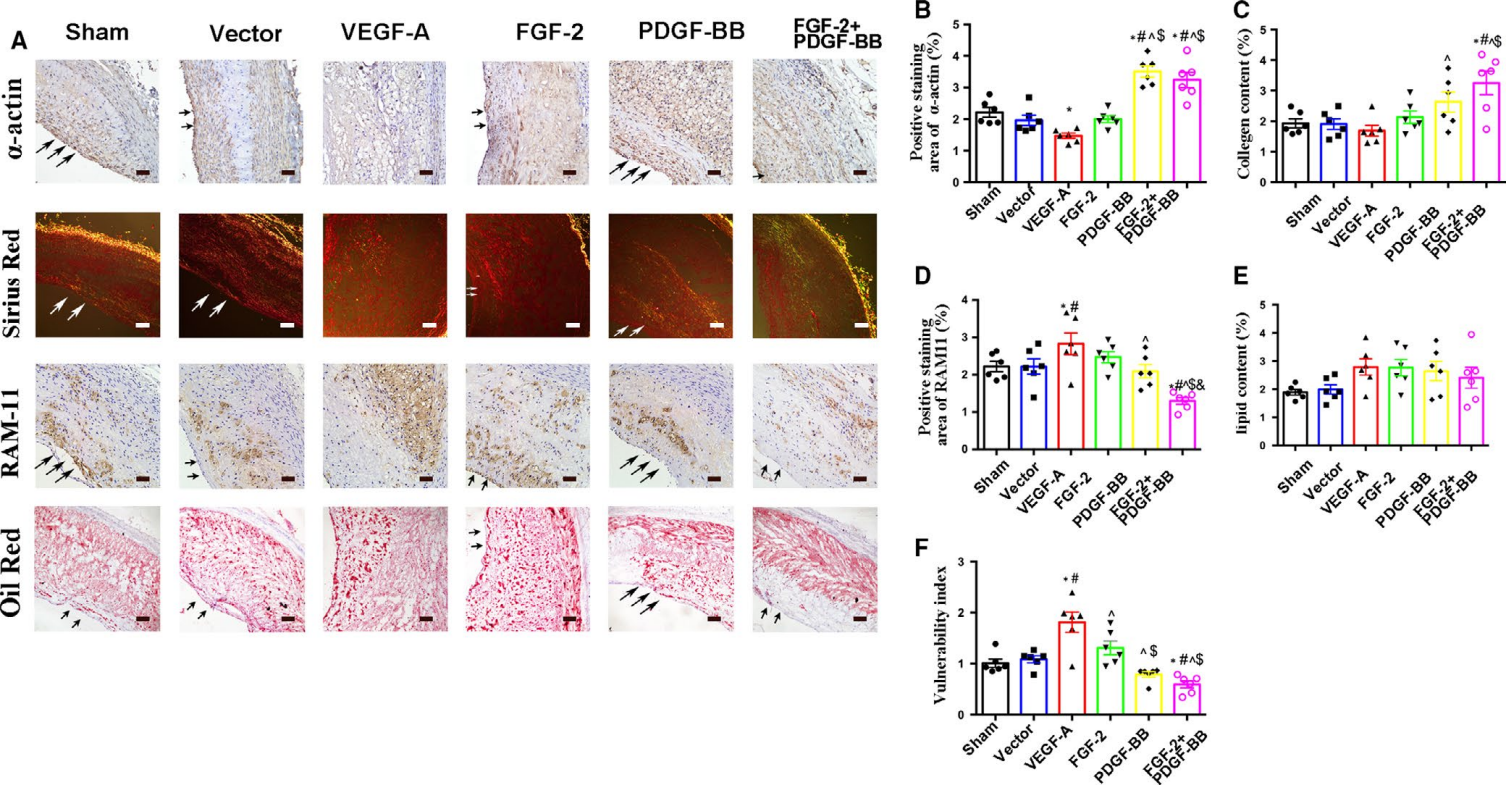
The effects of growth factors on atherosclerotic plaque vulnerability. A, Immunostaining of sections with the anti‐α‐SMC actin and anti‐RAM‐11 antibodies, Oil Red O staining and Sirius Red staining. Bar, 20 μm. B‐F, Quantification of α‐SMC+ and RAM‐11+ (macrophages) cells and quantification of Oil Red O+ (lipidosis) and Sirius Red+ (collagen) staining (mean ± SEM; n = 6). Arrow indicated lumen surface. ^*^ *P* < .05 vs Sham; ^#^ *P* < .05 vs Vector; ^^^ *P* < .05 vs VEGF‐A; ^$^ *P* < .05 vs FGF‐2; ^&^ *P* < .05 vs PDGF‐BB

To confirm plaque characteristics, plaques from all groups were evaluated by Masson trichrome, Oil Red O, RAM‐11 and α‐SMA actin staining. α‐SMA + staining in rabbits was significantly higher in the PDGF‐BB (3.46 ± 0.47%) and FGF‐2 + PDGF‐BB (3.28 ± 0.65%) groups than that in the VEGF‐A group (1.47 ± 0.22%, *P* < .05, Figure 2A,C). Additionally, collagen + staining was higher in the PDGF‐BB (2.75 ± 0.83%) and FGF‐2 + PDGF‐BB groups (3.18 ± 1.05% and 1.68 ± 0.48%, respectively*; P* < .05) than in VEGF‐A‐treated plaques (Figure 2A,D). Moreover, inflammatory macrophages infiltration at the sites of FGF‐2 + PDGF‐BB transfection was markedly decreased relative to that in the other groups (Figure 2A,E). These results demonstrated infiltration of a decreased number of inflammatory cells, increases in α‐SMA + and collagen + staining, and a decreased lipid content and necrotic area in stabilized FGF‐2 + PDGF‐BB‐treated plaques.

### 3.4 Combined FGF‐2 and PDGF‐BB induces much lower plaque neovessel density than FGF‐2 or PDGF‐BB used alone

**Figure 3:**
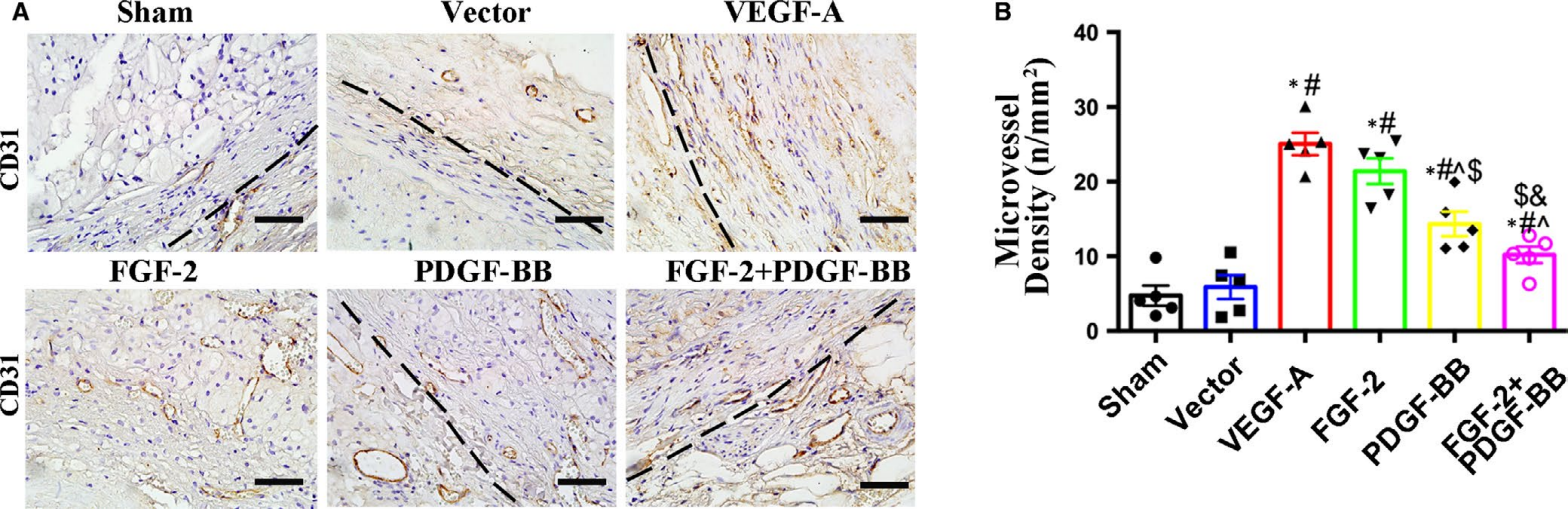
The effects of growth factors on intraplaque neovessels. A, Immunostaining of sections with the mouse anti‐CD31 antibody. Bar, 20 μm. B, Quantification of cells positive for mouse anti‐CD31 (circles indicate neovessels)/plaque area (mm^2^) (mean ± SEM; n = 6). Black dotted line indicates the boundaries between plaque and adventitia. ^*^ *P* < .05 vs Sham; ^#^ *P* < .05 vs Vector; ^^^ *P* < .05 vs VEGF‐A; ^$^ *P* < .05 vs FGF‐2; ^&^ *P* < .05 vs PDGF‐BB

To evaluate vascular remodelling associated with FGF‐2 + PDGF‐BB, we quantified the number of neovessels in plaques, revealing a 9‐, 8‐ and 5‐fold greater number of plaque neovessels in the VEGF‐A, FGF‐2 or PDGF‐BB groups, respectively, relative to that in the sham and vector groups (*P* < .05) (Figure 3A,B). Additionally, the number of plaque neovessels in FGF‐2 + PDGF‐BB‐treated lesions showed only a 3‐fold increase relative to those observed in the sham and vector groups (*P* < .05) (Figure 3A,B). These data demonstrated that combined FGF‐2 and PDGF‐BB treatment exerted a synergistic effect on inducing neovascularization within plaque, but that this effect was not as strong with VEGF‐A, FGF‐2 or PDGF‐BB treatment alone.

To determine why FGF‐2 and PDGF‐BB synergistically induce fewer neovessels than FGF‐2 alone, we investigated the plaque hypoxia level in each group. Atherosclerotic plaques develop intraplaque angiogenesis, which is a typical feature of hypoxia tissue. We used pimonidazole as a hypoxic probe to confirm the hypoxia state (Figure S3A). In the VEGF‐A group, hypoxia was 3‐ and 2‐fold higher than in the sham and vector groups, respectively, whereas in the FGF‐2 + PDGF‐BB group, the hypoxia level was much lower than that in the other groups (*P* < .05, Figure S3B). Based on these data, we explored protein level of HIF‐1α in each group (Figure S3C) finding that in the FGF‐2 + PDGF‐BB group, HIF‐1α levels were significantly reduced relative to those in the VEGF‐A and FGF‐2 groups (*P* < .05, Figure S3D).

### 3.5 Combined FGF‐2 and PDGF‐BB inhibits intraplaque haemorrhage (IPH) and erythrocyte extravasation

**Figure 4:**
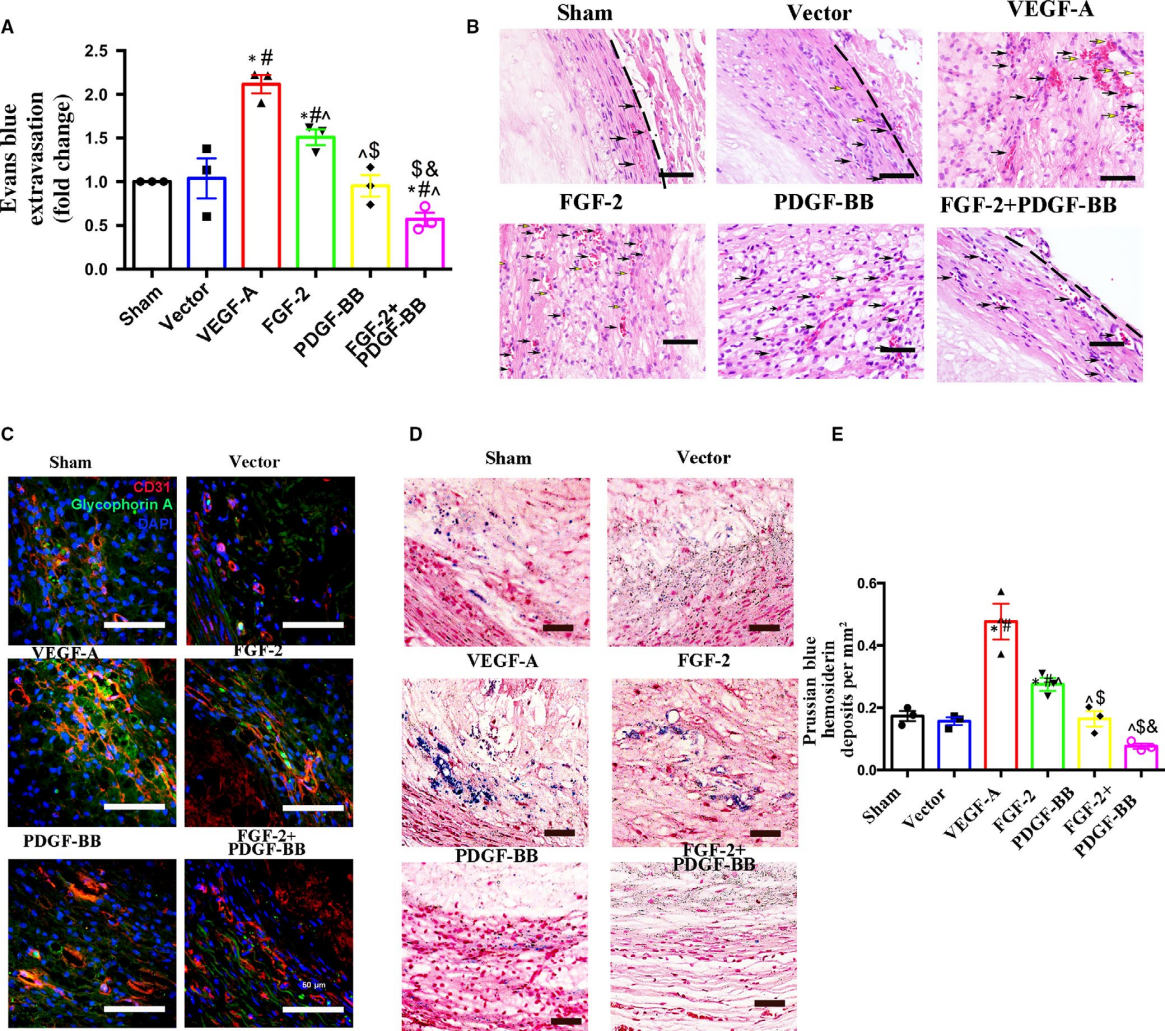
Histologic analysis of intraplaque neovessels in each group. A, Quantification of Evans Blue dye leakage into plaques in the sham, vector, VEGF‐A, FGF‐2, PDGF‐BB and FGF‐2 + PDGF‐BB groups (mean ± SEM; n = 3). B, H&E staining demonstrating the presence of RBCs (black arrows indicate RBCs within the luminal wall of neovessels and yellow arrows indicate RBC leakage from neovessels) within atherosclerotic plaques. Bar, 20 μm. C, Plaque sections from rabbit abdominal aortas from each group were analysed on week 8 after transfection by IF double labelling for CD31 (an endothelial‐cell marker; red) and glycophorin A (an erythrocyte marker; green). Bar, 50 μm. D, Representative bright‐field microscopy analysis of Prussian Blue + hemosiderin (blue) counterstained with Nuclear Fast Red (pink) in each group. E, Graph demonstrating quantification of Prussian Blue + hemosiderin deposits from the sham, vector, VEGF‐A, FGF‐2, PDGF‐BB and FGF‐2 + PDGF‐BB groups (mean ± SEM; n = 3). ^*^ *P* < .05 vs Sham; ^#^ *P* < .05 vs Vector; ^^^ *P* < .05 vs VEGF‐A; ^$^ *P* < .05 vs FGF‐2; ^&^ *P* < .05 vs PDGF‐BB

Vascular leakage is a key parameter used to monitor vascular function. Measurement of leakage by Evans Blue staining revealed that FGF‐2 + PDGF‐BB protected plaque neovessels from leakiness (Figure 4A). To explore the relationship between neovessel leakage and IPH, we examined IPH by H&E staining and glycophorin A staining. Almost no erythrocytes were observed outside the neovessels in the FGF‐2 + PDGF‐BB group, whereas in the VEGF‐A and FGF‐2 groups, we observed the presence of erythrocytes outside of the luminal areas of the neovessels (Figure 4B,C). Repeated IPH plays a major role in the evolution of plaque vulnerability and growth. After IPH, red blood cells (RBCs) rapidly release free haemoglobin, which can be phagocytosed or undergo rapid proteolysis, resulting in the release of free haem or free iron. Haem/iron can subsequently mediate oxidative modification of lipids and cause endothelial cytotoxicity.^18^ As revealed by Prussian Blue staining, the FGF‐2 + PDGF‐BB group displayed less iron content than the VEGF‐A, FGF‐2 and PDGF‐BB groups (*P* < .05) (Figure 4D,E). These results indicated that FGF‐2 and PDGF‐BB synergistically and significantly reduced IPH.

### 3.6 Combined FGF‐2 and PDGF‐BB augments pericyte coverage of plaque neovessels

**Figure 5:**
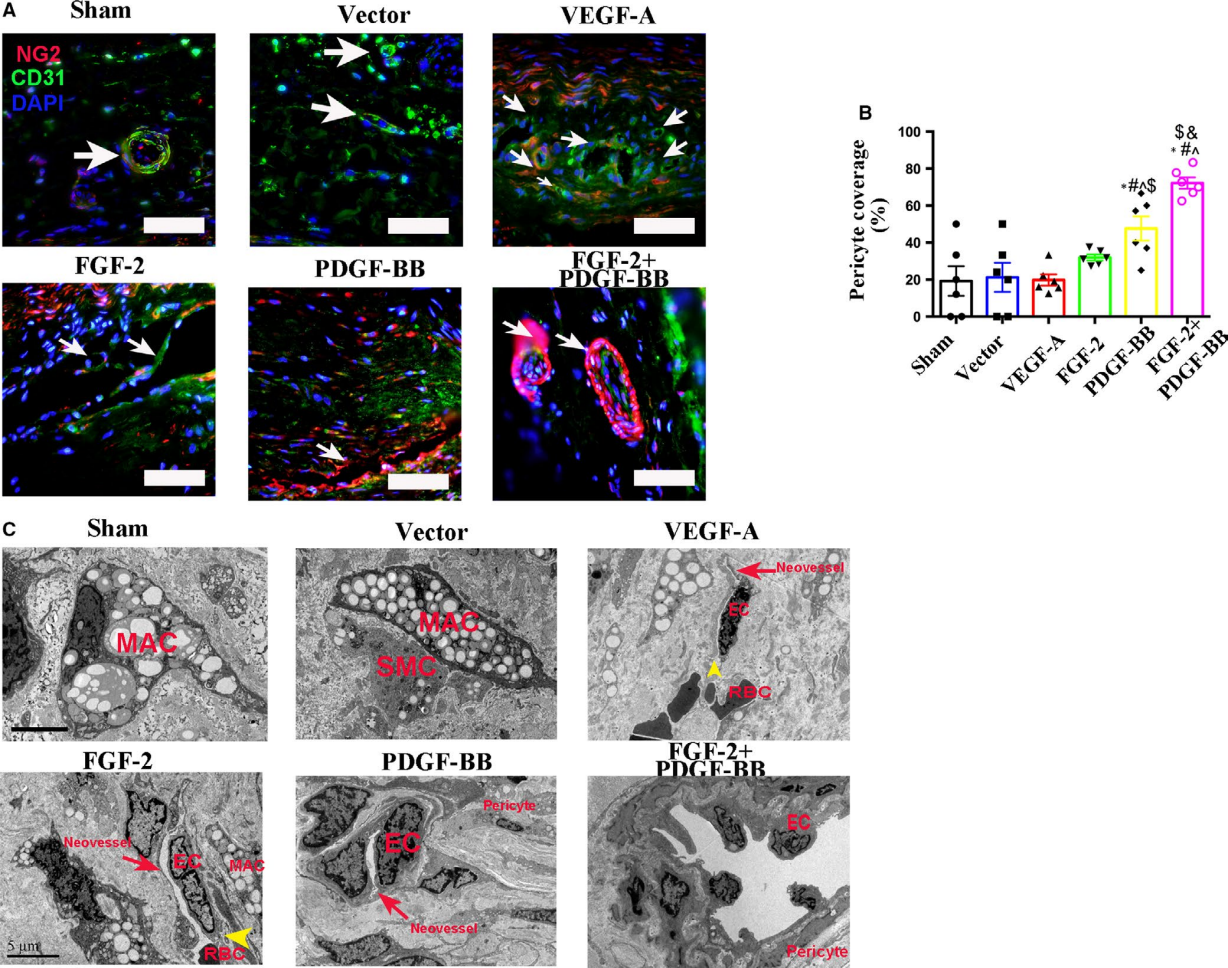
Analysis of pericyte coverage in the intraplaque neovessels of each group. A, Plaque sections from the sham, vector, VEGF‐A, FGF‐2, PDGF‐BB and FGF‐2 + PDGF‐BB groups were analysed on week 8 after transfection by IF double labelling for CD31 (an endothelial‐cell marker), NG2 (a pericyte marker) and autofluorescence (reddish yellow) of RBCs. Bar, 20 μm. Arrow indicated neovessels. B, Pericyte coverage (%) as percentages of pericyte + vessels in different groups. (n = 6 rabbits/condition). ^*^ *P* < .05 vs Sham; ^#^ *P* < .05 vs Vector; ^^^ *P* < .05 vs VEGF‐A; ^$^ *P* < .05 vs FGF‐2; ^&^ *P* < .05 vs PDGF‐BB. C, Plaque specimens were obtained from the sham, vector, VEGF‐A, FGF‐2, PDGF‐BB and FGF‐2 + PDGF‐BB groups and assessed by TEM for microstructural changes in the above organizations. Yellow arrow indicated the endothelial‐cell cleft. Bar, 5 μm. MAC, macrophage; SMC, smooth muscle cells; EC, endothelial cells; RBC, red blood cells

Pericytes play an essential role in blood‐vessel integrity and maturity. To assess the maturity of plaque neovessels induced by FGF‐2 + PDGF‐BB, we examined plaque sections for the expression of CD31 (an endothelial‐cell marker) and NG2 (a pericyte marker). Generally, pericyte circle around endothelial cells define mature neovessels in plaques. We found that the CD31 + NG2+ cell population was markedly increased in FGF‐2 + PDGF‐BB‐overexpressing plaques relative to that in control groups, suggesting that FGF‐2 + PDGF‐BB stimulated pericyte proliferation and recruitment to endothelial cells. By contrast, following induction with VEGF‐A or FGF‐2 alone, most intraplaque neovessels were immature and lacked pericyte coverage (Figure 5A). A maturation index (percentage of vessels coated with pericytes) used to assess neovessel maturity revealed that the index for FGF‐2 + PDGF‐BB‐induced vessels (~80%) was significantly higher than that for vessels induced with the other growth factors (Figure 5B).

We then examined inter‐endothelial junctions and pericyte coverage by TEM. Endothelial cells of the plaque neovessels exhibited blebbing and spike‐like protrusions of the cell membrane, with disconnection of the endothelial cells generally observed in the VEGF‐A and FGF‐2 groups (Figure 5C). By contrast, neovessels in the FGF‐2 + PDGF‐BB‐treated group showed endothelial junction integrity and pericyte coverage.

### 3.7 FGF‐2 + PDGF‐BB augments FGFR‐2, and PDGFR‐β expression associated with the plaques

**Figure 6:**
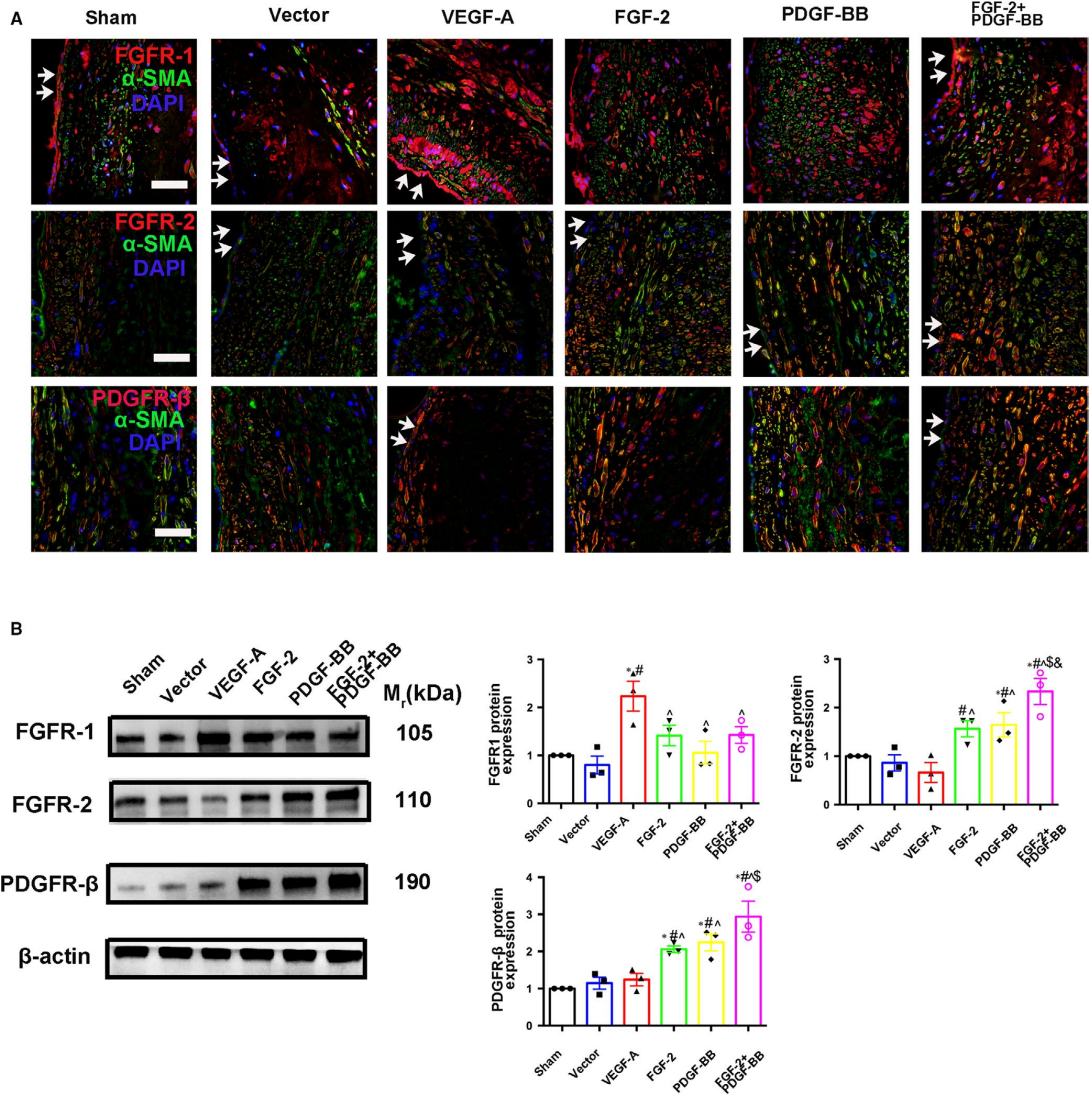
IF staining and Western blot for growth factors in rabbit aortic plaques. A, Immunofluorescence of sections from rabbit atherosclerotic plaques 8 wk after lentiviral transfection. Bar, 20 μm. Test the expression of FGFR‐1+, FGFR‐2+ and PDGFRβ+ cells in the sham, vector, VEGF‐A, FGF‐2, PDGF‐BB and FGF‐2 + PDGF‐BB groups (mean ± SEM; n = 6). Arrow indicated lumen surface. B, Western blot indicating protein levels of FGFR‐1, FGFR‐2 and PDGFRβ in the sham, vector, VEGF‐A, FGF‐2, PDGF‐BB and FGF‐2 + PDGF‐BB groups (mean ± SEM; n = 3). All data as standardized values. ^*^ *P* < .05 vs Sham; ^#^ *P* < .05 vs Vector; ^^^ *P* < .05 vs VEGF‐A; ^$^ *P* < .05 vs FGF‐2; ^&^ *P* < .05 vs PDGF‐BB

FGF‐FGFR and PDGF‐PDGFR systems are two characterized signalling pathways involved in pericyte recruitment in angiogenic vessels. To define the role of receptors associated with FGF‐2 + PDGF‐BB‐mediated neovessel maturation in plaques, we investigated the expression level of FGFR‐1, FGFR‐2 and PDGFR‐β in plaques. As expected, IF analysis following FGF‐2 + PDGF‐BB lentiviral transfection showed increased levels of FGFR‐2 and PDGFR‐β in the smooth muscle cells (Figure 6A), with elevated FGFR‐2 and PDGFR‐β protein levels in the FGF‐2 + PDGF‐BB group confirmed by Western blot (Figure 6B).

### 3.8 Combined FGF‐2 and PDGF‐BB augments pericyte migration in vitro

**Figure 7:**
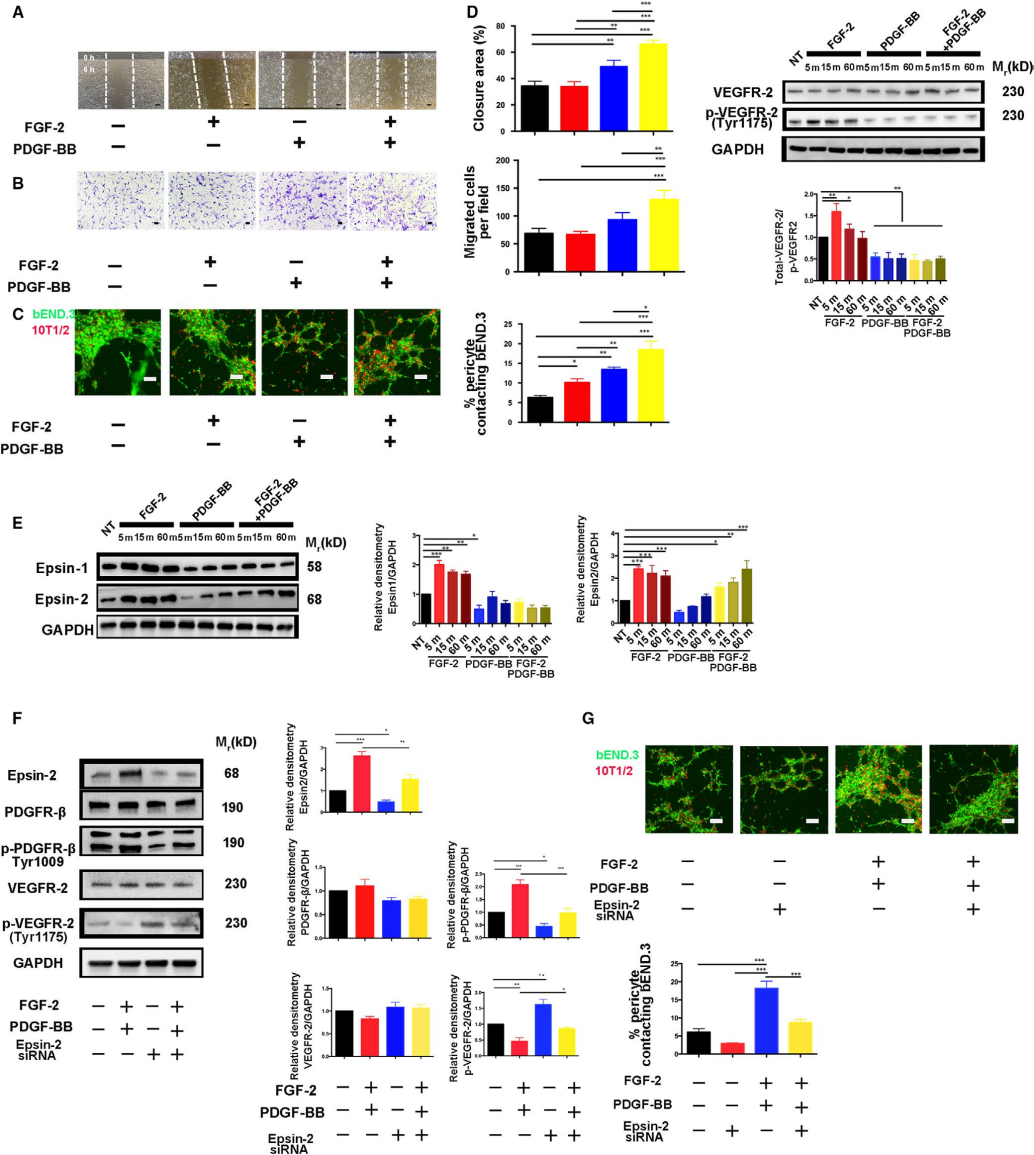
Effects of FGF‐2 + PDGF‐BB on pericyte migration, VEGFR2 degradation and epsin‐1/2 expression in vitro. A, Representative images and quantification of wound‐healing assay results at 0 and 6 h in FGF‐2‐, PDGF‐BB‐ or FGF‐2 + PDGF‐BB‐stimulated pericytes (n = 3 samples/group). Bar, 20 μm. B, Representative images and quantification of Transwell assays at 0 and 12 h in FGF‐2‐, PDGF‐BB‐ or FGF‐2 + PDGF‐BB‐stimulated pericytes (n = 3 samples/group). Bar, 20 μm. C, bEND.3 and 10T1/2 cells were labelled with carboxyfluorescein succinimidyl ester (green) and PKH26 (red), respectively and co‐cultured in Matrigel‐coated culture slides for 6 h under treatment with FGF‐2‐, PDGF‐BB‐ or FGF‐2 + PDGF‐BB. Bar, 20 μm. Quantification was performed by measuring the merged cells from 10 fields in three independent experiments. D, Time‐course analysis of total VEGFR‐2 and p‐VEGFR‐2 levels in FGF‐2‐, PDGF‐BB‐ or FGF‐2 + PDGF‐BB‐stimulated pericytes (n = 3 samples/group). E, Time‐course analysis of epsin‐1 and e‐2 protein levels in FGF‐2‐, PDGF‐BB‐ or FGF‐2 + PDGF‐BB‐stimulated pericytes (n = 3 samples/group). F, 10T1/2 cells transfected with either control siRNA or epsin‐2 siRNA were stimulated with FGF‐2 + PDGF‐BB and analysed by Western blot. Quantification of total PDGFR‐β, p‐PDGFR‐β, total VEGFR‐2 and p‐VEGFR2 levels (n = 3 samples/group). G, bEND.3 and 10T1/2 cells were labelled with carboxyfluorescein succinimidyl ester (green) and PKH26 (red), respectively, and co‐cultured in Matrigel‐coated culture slides for 6 h. Quantification was performed by measuring the merged cells from 10 fields in three independent experiments. Bar, 20 μm. All in vitro experiments were repeated at least twice. Data represent the mean ± SEM. All data as standardized values. *P < .05, **P < .01; and ***P < .001; analysis of variance. NT, no treatment

We then investigated the mechanisms underlying the potentiated biological functions of FGF‐2 + PDGF‐BB on pericytes in vitro using 10T1/2 cells as a pericyte model to evaluate the function of FGF‐2 + PDGF‐BB on pericyte recruitment to endothelial cells. As expected, FGF‐2 + PDGF‐BB induced pericyte migration (Figure 7A,B). We then used an in vitro cell‐recruitment assay to mimic pericyte recruitment and attachment to endothelial cells, revealing that 10T1/2 cell attachment to bEND.3 cells was significantly increased in the FGF‐2 + PDGF‐BB group as compared with other groups (*P* < .05) (Figure 7C).

### 3.9 FGF‐2 and PDGF‐BB enhance VEGFR2 degradation by increasing *epsin‐2* expression

A previous study reported VEGFR2 as a negative regulator of pericyte function^13^, ^21^; therefore, we examined VEGFR2 activation following stimulation with FGF‐2 + PDGF‐BB, finding that FGF‐2 + PDGF‐BB significantly decreased VEGFR2 phosphorylation (Figure 7D).

To identify the molecular mechanism underlying FGF‐2 + PDGF‐BB‐enhanced VEGFR2 degradation, we evaluated *epsin‐1/2* expression. Previous studies indicated that epsins regulate VEGFR2 signalling in endothelial cells^22^; however, their role in pericytes remains unknown. To investigate the role of epsins‐1 and ‐2 in pericytes, we examined their expression following stimulation of FGF‐2, PDGF‐BB and FGF‐2 + PDGF‐BB, respectively, in pericytes. As expected, FGF‐2 significantly increased *epsin‐1/2* expression in a time‐dependent manner, whereas FGF‐2 + PDGF‐BB treatment increased only *epsin‐2* expression in a time‐dependent manner (Figure 7E). Moreover, following siRNA‐mediated knock‐down of *epsin‐2* in pericytes, we observed augmented VEGFR2 signalling (Figure 7F). Additionally, cell‐recruitment assays showed that siRNA targeting *epsin‐2* markedly decreased the attachment of 10T1/2 cells to b.END3 cells (Figure 7G). These findings demonstrated that FGF‐2 + PDGF‐BB enhanced VEGFR2 degradation by increasing epsin‐2 expression.

## 4 DISCUSSION

Previous clinical observations indicated that plaque neovessel stabilization is therapeutically important. Although the harmfulness of immature intraplaque neovessels is well known, and current studies of vulnerable plaque generally focus on anti‐angiogenesis at the early stage of plaque formation and seldom directly address the ability of immature plaque neovessels to stabilize at an advanced stage of the plaque. This study revealed that overexpression of single angiogenic factors, including VEGF‐A, FGF‐2 and PDGF‐BB, was unable to establish stable and functional plaque neovessels, whereas combined overexpression of FGF‐2 and PDGF‐BB synergistically induced the formation of stable and functional neovessels. These results suggest a new therapeutic pathway for vulnerable plaque.

Here, we employed a rabbit model receiving media‐adventitia lentiviral injection to investigate the therapeutic potential of FGF‐2 + PDGF‐BB for increasing plaque neovessel maturity. FGF‐2 and PDGF‐BB synergistic effects have been investigated in cancers and some ischaemic diseases; however, the results were controversial.^23^, ^24^, ^25^ In rat and rabbit ischaemic hindlimb models, FGF‐2 and PDGF‐BB synergistically induced stable and relatively mature neovessels, but overexpression of FGF‐2 and PDGF‐BB in murine fibrosarcomas led to formation of high‐density primitive vascular plexuses that were poorly coated with pericytes and vascular SMCs.^23^, ^24^, ^25^ The plaque microenvironment is complex and a rich source of various signalling molecules; therefore, growth factors might exhibit different and unique biological functions in the plaque.^26^ In the present study, our initial observations suggested that the VEGF‐A and FGF‐2 groups significantly increased IPH and plaque rupture, whereas following FGF‐2 + PDGF‐BB overexpression, IPH and plaque rupture were seldom observed. VEGF‐A and FGF‐2 are potent angiogenic factors in vivo, and a recent study of ischaemic models showed that VEGF‐A‐ and FGF‐2‐overexpression induce primitive vascular plexuses.^23^ In the present study, VEGF‐A or FGF‐2 alone induced intense intraplaque angiogenesis, with these capillary networks usually comprising disorganized, leaky, premature and haemorrhagic blood vessels.

The newly formed blood vessels within FGF‐2 + PDGF‐BB‐overexpressing plaques were well organized and mature, with distinct vascular tree‐like structures and wide pericyte coverage. Pericytes are perivascular cells located at the interface between capillaries and surrounding tissues, where they play a key role in vascular maintenance and angiogenesis.^27^

The number of intraplaque neovessels induced by FGF‐2 + PDGF‐BB was significantly lower than that induced by either VEGF‐A‐ or FGF2‐overexpression separately. Oxygen deprivation in the plaque environment is a potent inducer of neovascularization; therefore, we investigated the plaque hypoxic state in each group, finding that the extent of plaque hypoxia was significantly reduced in the FGF‐2 + PDGF‐BB group. HIF‐1α is a potent inducer of neovascularization, and low levels might have provided negative feedback, resulting in a diminished number of intraplaque neovessels in the FGF‐2 + PDGF‐BB group.

The expression of PDGFRβ in plaque is a key event affecting the stability of intraplaque neovessels. Although PDGF‐BB overexpression increased PDGFRβ levels, as indicated according to CD31 and NG2 double staining, most of the neovessels in PDGF‐BB‐overexpressing plaques appeared as muscular circles consisting of only pericytes or vascular SMCs. This might be because PDGF‐BB is a potent mitogen that increases the migration and proliferation of PDGFR‐expressing vascular SMCs/pericytes while usually having no biological effect on endothelial cells not expressing detectable levels of PDGFRs.^28^ Intraplaque haemorrhage, hypoxia state and inflammatory cell infiltration were significantly increased in PDGF‐BB group. Therefore, newly formed vessels in the PDGF‐BB group were immature and leaky.

The mechanisms underlying pericyte recruitment induced by FGF‐2 and PDGF‐BB are complex. Previous studies demonstrated VEGFR‐2 as a negative regulator of pericyte function and PDGFRβ phosphorylation.^29^, ^30^ In the present study, we confirmed that FGF‐2 + PDGF‐BB enhanced VEGFR‐2 degradation in pericytes. Moreover, previous studies show that endothelial epsin‐1 and ‐2 play a key role in promoting VEGFR‐2 internalization and degradation.^22^, ^31^ In the present study, we showed that FGF‐2 + PDGF‐BB synergistically up‐regulated epsin‐2 as a mediator of VEGFR‐2 phosphorylation to enhance pericyte migration and recruitment to endothelial cells.

Although we have elucidated mechanisms involved in intraplaque neovessel normalization, future investigations should be focused on the following two aspects to facilitate efficient therapy of intraplaque angiogenesis. First, a nanoparticle delivery system should be implemented to achieve site‐specific delivery of FGF2 + PDGF‐BB, and concentration gradient studies should be conducted to ensure precise treatment of intraplaque angiogenesis. Second, the relationship between FGF‐2 + PDGF‐BB and epsin‐2 should be explored.

Altogether, our data provide new mechanistic insights into the signalling pathways associated with pericyte recruitment in plaque neovessels and suggest that drugs targeting these pathways would present a potential strategy for vulnerable plaque therapy.

## CONFLICT OF INTEREST

None.

## AUTHOR CONTRIBUTIONS

LZ and YZ conceived and designed the experiments. YM performed the experiments. YS analysed the data. CGZ, XLX and XQL contributed reagents/materials/analysis tools. YM contributed to the writing of the manuscript.

## Data Availability

The datasets used and analysed during the current study are available from the corresponding author on reasonable request.
