# Supplementary figures and images for: Fibroblast growth factor‐2/platelet‐derived growth factor enhances atherosclerotic plaque stability
Source: J Cell Mol Med. 2019 Nov 21;24(1):1128–40. doi: 10.1111/jcmm.14850 (PMC6933359; doi:10.1111/jcmm.14850)

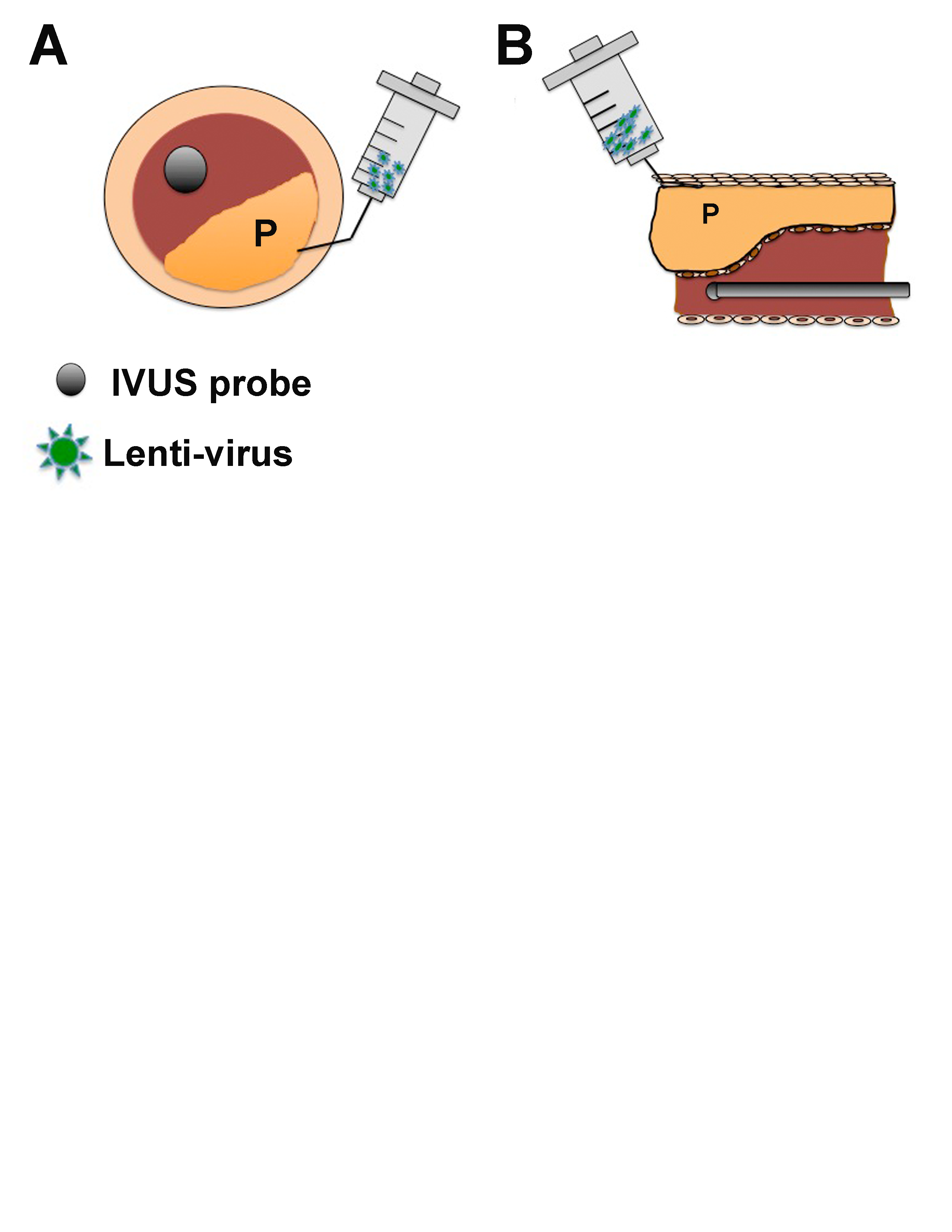

Supplement: Supplementary file 1 [file JCMM-24-1128-s001.tif]

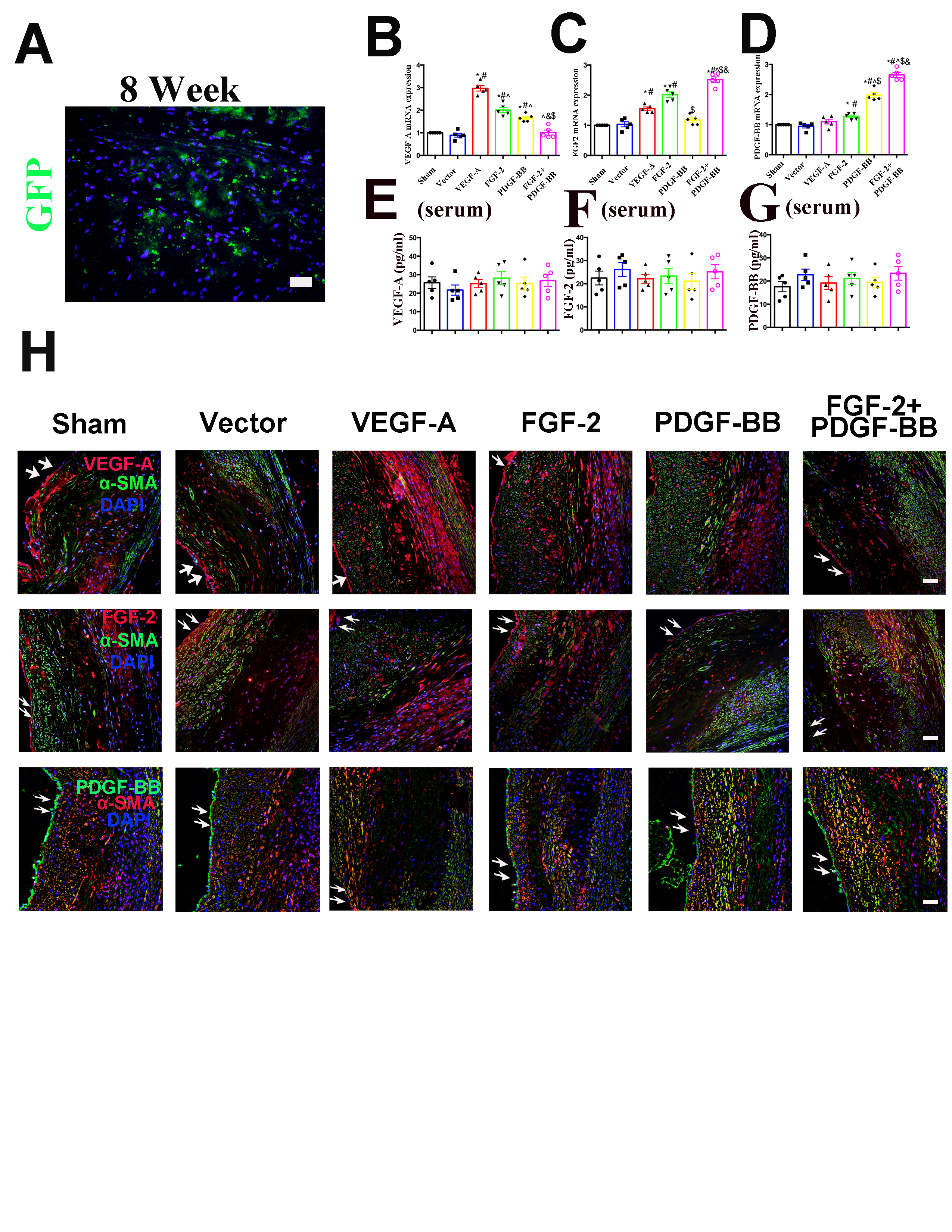

Supplement: Supplementary file 2 [file JCMM-24-1128-s002.tif]

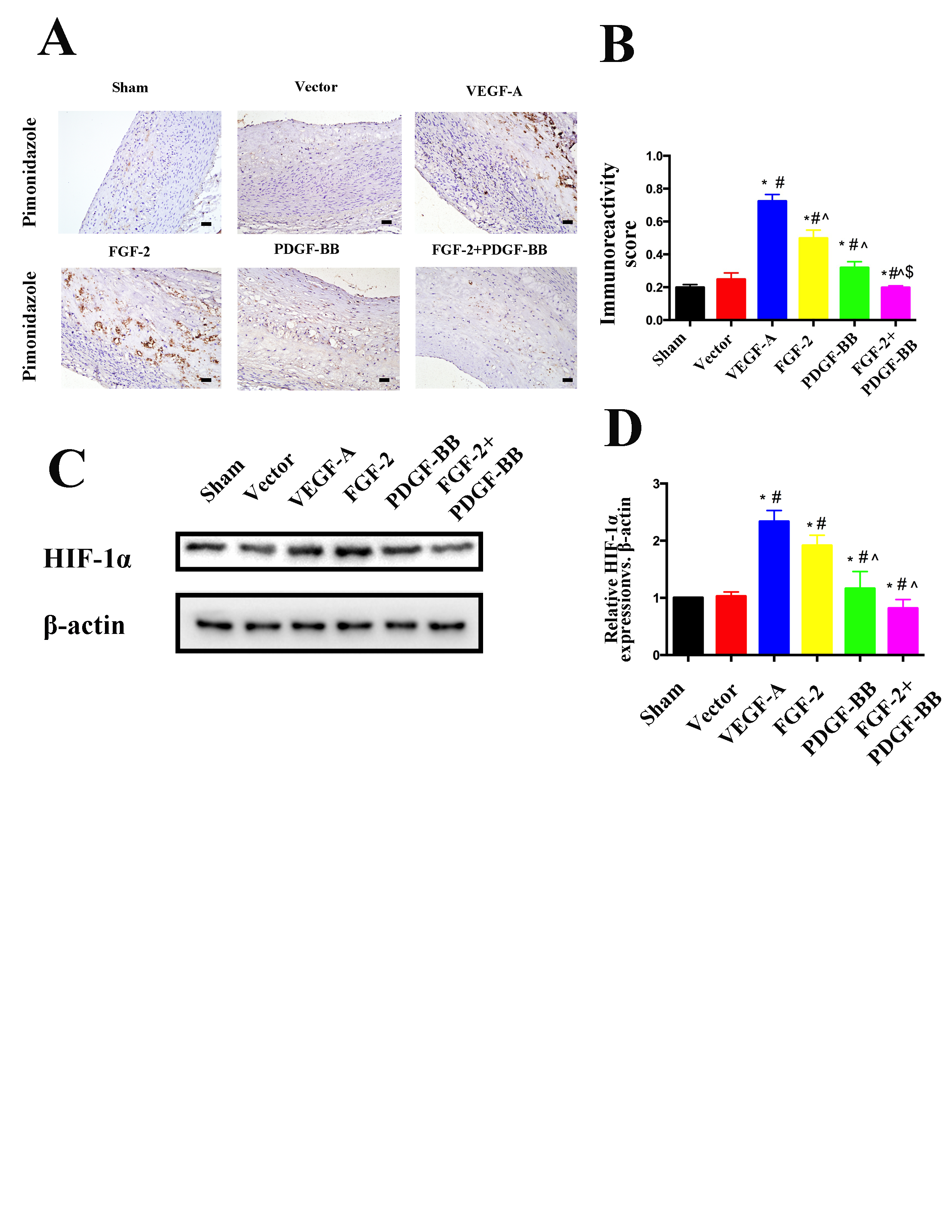

Supplement: Supplementary file 3 [file JCMM-24-1128-s003.tif]
