# Supplementary material for: Fibroblast growth factor‐2/platelet‐derived growth factor enhances atherosclerotic plaque stability
Source: J Cell Mol Med. 2019 Nov 21;24(1):1128–40. doi: 10.1111/jcmm.14850 (PMC6933359; doi:10.1111/jcmm.14850)
Supplement: Supplementary file 4 [file JCMM-24-1128-s004.docx]

**Supplement Table 1**

| **VEGF-A** |
| --- |
| ATGAACTT TCTGCTGT CTTGGGTG CATTGGAG CCTTGCCT TGCTGCTC TACCTCCA CCATGCCA AGTGGTCC CAGGCTGC ACCCATGG CAGAAGGA GGAGGGCA GAATCATC ACGAAGTG GTGAAGTT CATGGATG TCTATCAG CGCAGCTA CTGCCATC CAATCGAG ACCCTGGT GGACATCT TCCAGGAG TACCCTGA TGAGATCG AGTACATC TTCAAGCC ATCCTGTG TGCCCCTG ATGCGATG CGGGGGCT GCTGCAAT GACGAGGG CCTGGAGT GTGTGCCC ACTGAGGA GTCCAACA TCACCATG CAGATTAT GCGGATCA AACCTCAC CAAGGCCA GCACATAG GAGAGATG AGCTTCCT ACAGCACA ACAAATGT GAATGCAG ACCAAAGA AAGATAGA GCAAGACA AGAAAATC CCTGTGGG CCTTGCTC AGAGCGGA GAAAGCAT TTGTTTGT ACAAGATC CGCAGACG TGTAAATG TTCCTGCA AAAACACA GACTCGCG TTGCAAGG CGAGGCAG CTTGAGTT AAACGAAC GTACTTGC AGATGTGA CAAGCCGA GGCGGTGA |

| **FGF-2** |
| --- |
| ct ggtgggtgtg gggggtggag atgtagaaga tgtgacgccg cggcccggcg ggtgccagat tagcggacgc ggtgcccgcg gttgcaacgg gatcccgggc gctgcagctt gggaggcggc tctccccagg cggcgtccgc ggagacaccc atccgtgaac cccaggtccc gggccgccgg ctcgccgcgc accaggggcc ggcggacaga agagcggccg agcggctcga ggctggggga ccgcgggcgc ggccgcgcgc tgccgggcgg gaggctgggg ggccggggcc ggggccgtgc cccggagcgg gtcggaggcc ggggccgggg ccgggggacg gcggctcccc gcgcggctcc agcggctcgg ggatcccggc cgggccccgc agggaccatg gcagccggga gcatcaccac gctgcccgcc ttgcccgagg atggcggcag cggcgccttc ccgcccggcc acttcaagga ccccaagcgg ctgtactgca aaaacggggg cttcttcctg cgcatccacc ccgacggccg agttgacggg gtccgggaga agagcgaccc tcacatcaag ctacaacttc aagcagaaga gagaggagtt gtgtctatca aaggagtgtg tgctaaccgt tacctggcta tgaaggaaga tggaagatta ctggcttcta aatgtgttac ggatgagtgt ttcttttttg aacgattgga atctaataac tacaatactt accggtcaag gaaatacacc agttggtatg tggcactgaa acgaactggg cagtataaac ttggatccaa aacaggacct gggcagaaag ctatactttt tcttccaatg tctgctaaga gctga |

| **PDGF-BB** |
| --- |
| atgaatcg ctgctgggcg ctcttcctgt ctctctgctg ctacctgcgt ctggtcagcg ccgaggggga ccccattccc gaggagcttt atgagatgct gagtgaccac tcgatccgct cctttgatga tctccaacgc ctgctgcacg gagaccccgg agaggaagat ggggccgagt tggacctgaa catgacccgc tcccactctg gaggcgagct ggagagcttg gctcgtggaa gaaggagcct gggttccctg accattgctg agccggccat gatcgccgag tgcaagacgc gcaccgaggt gttcgagatc tcccggcgcc tcatagaccg caccaacgcc aacttcctgg tgtggccgcc ctgtgtggag gtgcagcgct gctccggctg ctgcaacaac cgcaacgtgc agtgccgccc cacccaggtg cagctgcgac ctgtccaggt gagaaagatc gagattgtgc ggaagaagcc aatctttaag aaggccacgg tgacgctgga agaccacctg gcatgcaagt gtgagacagt ggcagctgca cggcctgtga cccgaagccc ggggggttcc caggagcagc gagccaaaac gccccaaact cgggtgacca ttcggacggt gcgagtccgc cggcccccca agggcaagca ccggaaattc aagcacacgc atgacaagac ggcactgaag gagacccttg gagcctag |

VEGF-A, Vascular endothelial growth factor-A; FGF-2, Fibroblast growth factor; PDGF-BB, Platelet-derived growth factor
