# Supplementary material for: Fibroblast growth factor‐2/platelet‐derived growth factor enhances atherosclerotic plaque stability
Source: J Cell Mol Med. 2019 Nov 21;24(1):1128–40. doi: 10.1111/jcmm.14850 (PMC6933359; doi:10.1111/jcmm.14850)
Supplement: Supplementary file 5 [file JCMM-24-1128-s005.docx]

**Supplement Table 1. Primers for RT-PCR**

| **Primers** | **Sequences 5’-3’** |
| --- | --- |
| VEGF-A | Forward: TACCGTCTTCTTCCTCTGT |
|  | Reverse: GCCCTGGTGAGGTTTGAT |
| FGF-2 | Forward: GGGTTTCTTCCTGCGTAT |
|  | Reverse: CCAGTTCGTTTCAGTGCC |
| PDGF-BB | Forward: TGATCTCCAACGCCTGCT |
|  | Reverse: TCATGTTCAGGTCCAACTCG |
| GAPDH | Forward: CATCATCCCTGCCTCCACT |
|  | Reverse: GCCTGCTTCACCACCTTCTT |

VEGF-A, Vascular endothelial growth factor-A; FGF-2, Fibroblast growth factor; PDGF-BB, Platelet-derived growth factor
