# Supplementary material for: Fibroblast growth factor‐2/platelet‐derived growth factor enhances atherosclerotic plaque stability
Source: J Cell Mol Med. 2019 Nov 21;24(1):1128–40. doi: 10.1111/jcmm.14850 (PMC6933359; doi:10.1111/jcmm.14850)
Supplement: Supplementary file 6 [file JCMM-24-1128-s006.docx]

**Supplement Table 2.** Serum lipid profiles (mmol/L)

| **Paramete**r | **Group NC**  **(n=5)** | **Group GFP**  **(n=5)** | **Group VEGF-A**  **(n=5)** | **Group FGF-2**  **(n=5)** | **Group PDGF-BB**  **(n=5)** | **Group FGF-2+PDGF-BB**  **(n=5)** |
| --- | --- | --- | --- | --- | --- | --- |
| **HDL-C**  **(mmol/l)** | 1.225±0.136 | 1.395±0.145 | 1.373±0.117 | 1.304±0.150 | 1.267±0.137 | 1.234±0.059 |
| **LDL-C**  **(mmol/l)** | 2.268±0.226 | 2.098±0.180 | 2.039±0.119 | 2.137±0.150 | 1.966±0.129 | 2.169±0.193 |
| **TC**  **(mmol/l)** | 8.356±0.663 | 9.608±1.128 | 10.512±0.643 | 9.374±0.824 | 9.798±1.511 | 10.200±0.814 |
| **TG**  **(mmol/l)** | 0.680±0.039 | 0.760±0.060 | 0.778±0.028 | 0.733±0.036 | 0.700±0.064 | 0.778±0.090 |
| **BW**  **(kg)** | 3.317±0.140 | 3.244±0.111 | 3.222±0.204 | 3.290±0.177 | 3.438±0.181 | 3.269±0.368 |

Data are expressed as mean±SEM; *n*=no. of animals. HDL-C: high-density lipoprotein cholesterol; LDL-C: low-density lipoprotein cholesterol; TC: total cholesterol; TG: triglyceride.
