# Supplementary material for: Fibroblast growth factor‐2/platelet‐derived growth factor enhances atherosclerotic plaque stability
Source: J Cell Mol Med. 2019 Nov 21;24(1):1128–40. doi: 10.1111/jcmm.14850 (PMC6933359; doi:10.1111/jcmm.14850)
Supplement: Supplementary file 7 [file JCMM-24-1128-s007.docx]

**Supplementary Figure 1. Surgical diagram.** (A) Cross-section of plaques to show the method of media-adventitia injection of lenti-virus. (B) Longitudinal section of plaque to show the method of media-adventitia injection of lenti-virus.

**Supplementary Figure 2. Verify transfection efficiency.** (A) Representative images of frozen plaque sections corresponding to 8-week post-GFP lentiviral transfection. (B-D) qRT-PCR analysis of relative mRNA levels of VEGF-A, FGF-2, and PDGF-BB in aortic plaques from each group (mean ± SEM; *n* = 5). (E-G) ELISA analysis of relative serum levels of VEGF-A, FGF-2, and PDGF-BB from each group (mean ± SEM; n = 5). (H) Immunofluorescent (IF) staining assay of VEGF-A (Abcam, ab1316) and α-SMA (19245, Cell Signaling Tchonology) co-location, FGF-2 (Abcam, ab181) and α-SMA (19245, Cell Signaling Tchonology) co-location, and PDGF-BB (Abcam, ab178409) and α-SMA (Abcam, ab7817) co-location within the plaques from each groups. ^*^*P*<0.05 vs. group Sham; ^#^*P*<0.05 vs. group Vector; ^^^*P*<0.05 vs. group VEGF-A; ^$^*P*<0.05 vs. group FGF-2; ^&^*P*<0.05 vs. group PDG-BB.

**Supplement Figure 3. The effects of growth factors on plaque hypoxia state.** **(A-B)** Representative pictures and quantification of pimonidazole (borrow) in aortic plaques from *group Sham*, *group Vector*, *group VEGF-A*, *group FGF-2*, *group PDGF-BB* and *group FGF-2+PDGF-BB*. (Bar=20μm). **(C-D)** Western blot analysis and quantification for HIF-1α. (mean±SEM *n*=3). ^*^*P*<0.05 vs. group Sham; ^#^*P*<0.05 vs. group Vector; ^^^*P*<0.05 vs. group VEGF-A; ^$^*P*<0.05 vs. group FGF-2; ^&^*P*<0.05 vs. group PDG-BB.
